# An implementation framework for evaluating the biocidal potential of essential oils in controlling *Fusarium* wilt in spinach: from *in vitro* to *in planta*

**DOI:** 10.3389/fpls.2024.1444195

**Published:** 2024-08-06

**Authors:** Mahyar Mirmajlessi, Neda Najdabbasi, Loredana Sigillo, Geert Haesaert

**Affiliations:** ^1^ Department of Plants and Crops, Ghent University, Faculty of Bioscience Engineering, Ghent, Belgium; ^2^ Council for Agricultural Research and Economics (CREA), Research Centre for Vegetable and Ornamental Crops, Pontecagnano, Italy

**Keywords:** anti-fungal activity, disease management, *Fusarium oxysporum* f.sp. *spinaciae*, fusarium wilt, plant-based compounds

## Abstract

*Fusarium* wilt, caused by *Fusarium oxysporum* f. sp. *spinaciae*, causes a significant challenge on vegetative spinach and seed production. Addressing this issue necessitates continuous research focused on innovative treatments and protocols through comprehensive bioassays. Recent studies have highlighted the potential of plant-based compounds in controlling fungal diseases. The present work aims to conduct a series of experiments, encompassing both *in vitro* and *in planta* assessments, to investigate the biocontrol capabilities of different essential oils (EOs) at various application rates, with the ultimate goal of reducing the incidence of *Fusarium* wilt in spinach. The inhibitory effect of four plant EOs (marjoram, thyme, oregano, and tea tree) was initially assessed on the spore germination of five unknown *Fusarium* strains. The outcomes revealed diverse sensitivities of *Fusarium* strains to EOs, with thyme exhibiting the broadest inhibition, followed by oregano at the highest concentration (6.66 μL/mL) in most strains. The tested compounds displayed a diverse range of median effective dose (ED50) values (0.69 to 7.53 µL/mL), with thyme and oregano consistently showing lower ED50 values. The direct and indirect inhibitory impact of these compounds on *Fusarium* mycelial growth ranged from ~14% to ~100%, wherein thyme and oregano consistently exhibiting the highest effectiveness. Following the results of five distinct inoculation approaches and molecular identification, the highly pathogenic strain F-17536 (*F. oxysporum* f.sp. *spinaciae*) was chosen for *Fusarium* wilt assessment in spinach seedlings, employing two promising EO candidates through seed and soil treatments. Our findings indicate that colonized grain (CG) proved to be a convenient and optimal inoculation method for consistent *Fusarium* wilt assessment under greenhouse conditions. Seed treatments with thyme and oregano EOs consistently resulted in significantly better disease reduction rates, approximately 54% and 36% respectively, compared to soil treatments (P > 0.05). Notably, thyme, applied at 6.66 µL/mL, exhibited a favorable emergence rate (ERI), exceeding seven, in both treatments, emphasizing its potential for effective disease control in spinach seedlings without inducing phytotoxic effects. This study successfully transitions from *in vitro* to *in planta* experiments, highlighting the potential incorporation of EOs into integrated disease management for *Fusarium* wilt in spinach production.

## Introduction

Fungal diseases caused by *Fusarium* species cause significant economic threats, contributing to a wide range of plant diseases such as damping off, root rot, stem rot, leaf spots, crown rot, and vascular wilts, thereby posing challenges to crops cultivation worldwide. Furthermore, the secondary metabolites produced by the genus *Fusarium*, known as mycotoxins, exhibit a broad range of toxic effects, leading to both acute and chronic diseases in animals and humans (Antonissen et al., 2014). Over 50 species of *Fusarium* have been identified, encompassing both plant and animal pathogens, but only a few of these species are known to cause infections in humans (Nucci and Anaissie, 2007). Spinach (*Spinacia oleracea* L.) is prone to various diseases, leading to reduced production worldwide. Amongst them, *Fusarium* wilt, caused by the fungus *Fusarium oxysporum* f. sp. *spinaciae*, is recognized as the most prevalent and severe disease of spinach. It leads to symptoms ranging from seed decay, wilting, stunted growth, and discoloration of the vascular system to damping-off and plant death, in both young seedlings and fully grown plants (Mowlick et al., 2013; Ekwomadu and Mwanza, 2023). This disease is also a concern in spinach seed production because the pathogen is readily seedborne and can spread through soil, enabling its survival for very long periods even in the absence of host plants (Larena et al., 2003; Sharma et al., 2017). Additionally, the long-lasting chlamydospores, which can persist for over 10 years, pose a significant challenge in controlling *Fusarium* wilt disease (Egel and Martyn, 2007; Bahadur, 2022). Despite the development of various control strategies, their effectiveness in controlling *Fusarium* diseases has been insufficient. Specifically, the use of synthetic fungicides to fight *Fusarium* wilt has been discouraged as they can harm soil health and potentially affect humans and other non-target organisms in the environment (De Cal et al., 2009; Sharma et al., 2017). Hence, exploring potent bioactive compounds for controlling *Fusarium* diseases directly or in combination with other strategies has become the new emphasis.

Plant-derived bioactive compounds are highlighted for their environmental safety, as they are biodegradable and exhibit minimal to no toxicity towards mammals and non-target organisms (Linde et al., 2010; Kumar et al., 2014; Sharma et al., 2017; Moungthipmalai et al., 2023). Essential oils (EOs), derived from organic sources, offer a range of benefits such as low residue levels, broad-range antimicrobial effectiveness and diverse mechanisms of action, making them promising eco-friendly green preservative agents and alternatives to synthetic fungicides (Han et al., 2019; Najdabbasi et al., 2020; Prakash et al., 2024). They possess remarkable permeability and high volatility, and are composed of a diverse array of secondary metabolites obtained from aromatic plants. Indeed, aromatic plants are abundant sources of bioactive compounds, including tannins, sterols, carvacrol, flavonoids, phenols, alkaloids, quinones, and saponins (Khan et al., 2015; Han et al., 2019). These compounds have demonstrated strong antifungal impacts by affecting cell wall/membrane, inhibiting quorum sensing, altering hyphal morphology, preventing biofilm formation, and suppressing mycotoxin synthesis/production (Chen et al., 2013; Sardi et al., 2014; Haque et al., 2016; Najdabbasi et al., 2020; Abdi-Moghadam et al., 2023). EOs may exert their effects through the active components, either directly influencing the pathogen or inducing systemic resistance in host plants, ultimately leading to a reduction in disease development. Previous studies have reported the antifungal activity of different EOs against various phytopathogens, such as *Didymella bryoniae, Colletotrichum lupini, C. lindemuthianum, Penicillium digitatum*, *Botrytis cinerea, Pleiochaeta setosa, Rhizoctonia solani, Sclerotinia sclerotiorum, Pythium ultimum* and *Fusarium* spp (Fiori et al., 2000; Soylu et al., 2007; Sharma et al., 2017; Dewitte et al., 2018; Bounar et al., 2020; Najdabbasi et al., 2020; Khaleil et al., 2021; Parikh et al., 2021). Many EOs and their major components are classified as “generally recognized as safe” (GRAS) by regulatory authorities such as the Food and Drug Administration (FDA) and the Environmental Protection Agency (EPA), underlining their safety and highlighting their potential effectiveness in combatting fungal pathogens (Tolouee et al., 2010; Lu et al., 2013). Besides extensive research on various target pathogens, the potential of EOs to be used in combination with other EOs or biocontrol agents (Abdel-Kader et al., 2011; Tejeswini et al., 2014; Pane et al., 2017; Zimmermann et al., 2023) makes them promising for controlling *Fusarium* diseases. In addition, given the modest biological activity of individual components and the synergistic or antagonistic interactions among them, it is important to consider the entire EO for disease management rather than isolating and synthesizing individual components (Bi et al., 2012; Stevic et al., 2014; Nazzaro et al., 2017; Perczak et al., 2019). Despite these, the existing literature on the utilization of EOs for controlling *Fusarium* wilt in spinach is currently limited. Similarly, there is less information available on the efficiency of artificial inoculation methods for pathogenicity assessment of *Fusarium oxysporum* f. sp. *spinaciae*, thereby establishing an optimal inoculation technique is crucial in evaluating the effectiveness of biological control strategies.

Consequently, the objectives of this study were i) to assess the potential of pure EOs as a safe and environmentally-friendly alternative to synthetic chemicals. This encompassed a series of *in vitro* bioassays that examined the fungitoxicity of EOs on spore germination and mycelial growth of *Fusarium* strains; ii) to compare various pathogenicity tests and determine the optimal inoculation method for assessing disease severity of *Fusarium oxysporum* f. sp. *spinaciae* on spinach; and iii) to investigate the efficacy of chosen EOs in reducing *Fusarium* wilt of spinach through *in planta* bioassays.

## Materials and methods

### Fungal isolates, inoculum preparation and plant EOs

A total of 11 unknown *Fusarium* strains were isolated from various host plants and diverse geographical locations. Among these, eight were obtained from Enza Zaden (Vegetable-Breeding Company) in the Netherlands, and three from the Research Centre for Vegetable and Ornamental Crops (CREA) in Italy. However, only five strains out of the total of eleven were randomly selected for use in the *in vitro* experiments, with selection criteria based on their respective host plants and geographical origins. To prepare spore suspensions, each isolate was sub-cultured onto a Potato Dextrose Agar (PDA, 39 gr/L) (Sigma Aldrich, Overijse, Belgium) plate and incubated at 25°C in darkness for 5 days. The mature mycelium was then transferred to fresh PDA plates and subjected to near-ultraviolet (NUV) light and dark cycles, following a 12-hour photoperiod, for 10 days to induce sporulation (International Seed quality Assurance (ISTA) (2023). Afterward, the mycelial surface was gently rubbed with 10 mL of sterile distilled water (SDW), and the resulting mixture was filtered through a sterilized Mira-cloth layer (Merck, Darmstadt, Germany). The spore suspensions were quantified using a hemocytometer and diluted to a concentration of 5 × 10^5^ conidia/mL. Five commercially pure pharmaceutical grade EOs (Omega Pharma, Phytosun aroms, France) including tea tree (*Melaleuca alternifoliai*), thyme (*Thymus vulgaris*), oregano (*Origanum vulgare*) and marjoram (*Origanum majorana*) were examined for their antifungal activity against *Fusarium* strains. The EOs were stored at low temperature (4°C) and kept protected from light and humidity for future use. To obtain suitable preparations, the aforementioned EOs were dissolved in SDW at the following concentrations: 0.83, 1.66, 3.33, and 6.66 μL/mL, with 0.1% Tween 80 (Sigma-Aldrich) added.

### Inhibitory effects of EOs on spore germination

#### Agar well diffusion method

For the initial screening, the standard well diffusion assay was employed by incorporating each EO into molten PDA (at approximately 50°C) to assess their antifungal activity against five *Fusarium* strains. For each strain, spore suspension (5 × 10^5^ conidia/mL) was dispensed onto the surface of solidified PDA plate. Following a drying period of approximately 5 minutes, two wells with a diameter of 6 mm were created on each agar plate (90 mm) using a sterile cork-borer. These wells were then filled with 100 µL of EOs at different concentrations (0.83, 1.66, 3.33, and 6.66 µL/mL). Subsequently, the culture plates were incubated at 25°C for 10 days, and screened for the presence of a zone of inhibition, also known as the zone of clearance. The effectiveness of the tested EOs activity was obtained based on diameter of zone of inhibition (with 6 mm of the well) around the well.

#### Microtiter plate method

The inhibitory effect of EOs at various concentrations on the spore germination of *Fusarium* species was assessed using a 96-well microtiter plate assay, based on photometric measurements, following a previously described method (Najdabbasi et al., 2020). In each well of the microtiter plate, a mixture of 20 μL of the respective compound’s concentrations, 180 μL of potato dextrose broth (Potato dextrose broth, 24 g/L) (Sigma Aldrich, Overijse, Belgium), and 20 μL of spore suspension (5 × 10^5^ conidia/mL) was added. The negative control wells in each replicate block contained 20 μL of water instead of the spore suspension. After 8 days of incubation, spore germination in each plate was quantified using a microplate photometer (Asys UVM 330 Plate Reader) to measure the optical density at 620 nm (OD 620). Consequently, the median effective dose (ED50) was derived from the net OD, representing the point at which spore germination was reduced by 50% compared to the control.

### Inhibitory effects of EOs on mycelial growth

#### Agar dilution method

The mycelial growth of *Fusarium* strains was evaluated through a dose-response assay using petri plates containing PDA media supplemented with different concentrations of the EOs. Mycelial plugs (6 mm) of fungal isolates were taken from the actively growing edges of the cultures and transferred to the center of each pre-amended PDA petri plate. The control comprised PDA without any EO. Following incubation in darkness at 25°C, the average radial growth was determined by measuring the diameters of the colonies in two perpendicular directions using a digital caliper (Solna, Sweden) every two days, until the mycelia fully covered the medium in the control petri plate. Consequently, the inhibitory percentage of different concentrations of EOs was calculated using Abbott’s formula (Abbott, 1925):


$$MI\;\left(\%\right)\;=\frac{C-T}{C}\;X\;100$$

wherein, MI represents the percentage of mycelial growth inhibition, and C and T refer to the diameter of the fungus colony (mm) in the control and the relevant treatment, respectively. Furthermore, the minimum inhibitory concentration (MIC) and the minimum fungicidal concentration (MFC) of each EO were determined by sub-culturing the inhibited fungal plugs from the EO-treated plates to newly prepared PDA plates without EO (Najdabbasi et al., 2020). After incubation for an additional seven days at 25°C, the lowest EO concentration that inhibited mycelial growth but allowed growth revival upon transfer was identified as the MIC. Conversely, the lowest EO concentration that prevented mycelial growth after transfer to fresh EO-free PDA plates was recognized as the MFC.

#### Volatile method

The impact of the volatile compounds from the same EOs on the mycelial growth of *Fusarium* strains was also evaluated at different concentrations, according to a previously reported method (Parikh et al., 2021). Briefly, a mycelial plug (6 mm) obtained from a 10-day-old culture grown on PDA was transferred to the center of a new PDA plate. A volume of 10 µl of the EOs was applied to the inner side of the inverted lid of the fresh PDA plate, and then the plate containing the mycelial plug was inverted and placed onto the lid. The control plates were left untreated without any addition of EO. To prevent any leakage of the active components, the petri plates were tightly sealed with parafilm. The percentage of mycelial growth inhibition was determined for each treatment by measuring the colony diameter of each pathogen 10 days after inoculation, as previously described. Furthermore, the fungal plugs from treatments showing no fungal growth were transferred to untreated PDA culture medium and placed in an incubator at 25°C for an additional week for further evaluation to ascertain fungistatic or fungicidal activity of each EO, as described earlier. The *in vitro* bioassays were conducted twice, and each treatment was subjected to six replications. Following the evaluation of results obtained from *in vitro* bioassays, the EO that exhibited the most effective antifungal activity against *Fusarium* strains at its applied concentrations was chosen for subsequent investigation in *in vivo* bioassays.

### Molecular identification

The subsequent molecular analysis aimed to confirm the identity of *Fusarium* strains, which was critical for designing the subsequent *in planta* experiments targeting disease control. The identification process was carried out for all 11 unidentified strains. Total genomic DNA was extracted from mycelia cultured in PDB for seven days at 22°C using a DNeasy Plant Mini Kit (Qiagen, USA) according to the manufacturer’s instructions. The identification of each fungus involved amplification of the internal transcribed spacer (ITS) region (*ITS1-5.8SITS4*) of the ribosomal RNA (rRNA) using primers ITS1 (5’-TCCGTAGGTGAACCCTGCGG-3’) and ITS4 (5’-TCCTCCGCTTATTGATATGC-3’) (White et al., 1990), along with amplification of the elongation factor (*TEF-1α*) using primers EF-1α (5’-ATGGGTAAGGAAGACAAGAC-3’) and EF2 (5’-GGAAGTACCAGTGATCATGTT-3’) (O’Donnell et al., 1998). For PCR, all samples were amplified with a final volume of 25 μl, consisting of 12.5 μl of GoTaq^®^ Colorless Master Mix (Promega Corporation, Madison, WI), 0.25 μl each of 50 μM forward and 50 μM reverse primers, 2 μl of DNA template, and 10 μl of nuclease-free water, and the amplification was performed using the GeneAmp PCR system 97,000 PCR (Applied Biosystem, Foster City, CA, USA). The amplification parameters for ITS1/4 included an initial denaturation step at 94°C for 5 min, followed by 30 cycles of denaturation at 94°C for 40 s, annealing at 58°C for 40 s, and elongation at 72°C for 40 s, with a final extension step at 72°C for 5 min. The amplification conditions for EF1/2 involved an initial denaturation at 94°C for 5 min, followed by 33 cycles of denaturation at 94°C for 30 s, annealing at 51°C for 30 s, elongation at 72°C for 30 s, and a final extension at 72°C for 5 min. After PCR amplification, the amplicons were separated on a 1% (w/v) agarose gel, stained with ethidium bromide for 30 minutes, and visualized using the Molecular Imager^®^ Gel Doc™ XR+ System with Image Lab™ Software (BIO-RAD, Hercules, CA, USA). Following that, the amplicons were purified using the E.Z.N.A.^®^ Cycle-Pure Kit (VWR International, Leuven, Belgium), and the resulting purified PCR products were sent to LGC Genomics (LGC group, Berlin, Germany) for Sanger sequencing. Identification of *Fusarium* strains was accomplished by performing a BLAST-search on NCBI (National Center for Biotechnology Information) database.

### Assessments of the antifungal activity of EOs - in planta

#### Inoculation method

Following molecular identification, various inoculation methods were performed on a single strain of *Fusarium* species isolated from spinach host plant. This aimed to identify the most effective and efficient assessment method suitable for subsequent greenhouse experiments. To set up plant materials, spinach seeds were placed on two layers of blotting paper in petri plates, and subsequently transplanted into standard potting soil after germination. In the first pathogenicity test, the soil drench (SD) method, the fungus spore suspension (5 × 10^5^ conidia/mL) was drenched using a repeater syringe onto the root plugs of two-week-old spinach seedlings (var. Crosstrek F1) grown in 0.5 L plastic pots containing standard potting soil (organic/mineral fertilizers NPK 14-16-18, pH (H2O) 5–6.5, organic and dry matters: 25% and 20%, respectively), with three seedlings per pot. In pathogenicity test 2, the root-dip inoculation (RD) method, the bottom 20% of the root plugs of two-week-old seedlings were cut manually to provide wounding for infection, then swirled in 100 ml of the fresh spore suspension for two minutes. Inoculated seedlings were then transplanted into plastic pots containing standard potting soil. The pathogenicity test 3, seed soaking (SS) method, involved soaking spinach seeds in spore suspension for one hour. Following this soaking period, the seeds were allowed to dry before being planted into the soil. In pathogenicity test 4, the mycelial plug (MP) inoculation method, seedlings roots were inoculated with fungal mycelial plugs. Two weeks after transplantation, the root zones of the seedlings were inoculated with 6 mm fungal mycelial plugs. In pathogenicity test 5, the colonized grain (CG) method, spinach seeds were planted in pots filled with the mixture of standard potting soil and ground wheat grains colonized by the fungus at a ratio of 10 g inoculum/L of substrate. *Fusarium* strain had been grown on sterilized wheat grains at 25°C for two weeks. Throughout all tests, a non-inoculated treatment was also included in each pathogenicity test as a negative control. Pots were maintained in the growth chamber at 21°C and 12 h photoperiod for four weeks. A rating scale was developed to qualitatively determine the disease severity index (DSI) based on visual foliar symptoms, ranging from 0 to 3: 0 for a healthy plant, 1 for slight wilting, 2 for severe wilting, and 3 for a dead plant. The pathogenicity tests were conducted twice with 10 pots per treatment. The assessment was conducted on a percentage basis using the following formula:


$$DSI\;\left(\%\right)\;=\;\left[\frac{\Sigma \left(class\;occurrence\;\times \;grading\;class\;score\right)}{\left(total\;plants\;in\;a\;set\right)\;\times \left(maximum\;disease\;grading\right)}\;\right]X\;100$$

### Phytotoxic effect assessment

The phytotoxicity of the tested oils on spinach seeds was also assessed using a germination test following the standard “top of paper method” detailed in the International Seed Testing Association (ISTA), with slight modifications. Two EOs, at concentrations of 3.33 and 6.66 µL/mL, were chosen due to their superior efficacy observed across all strains *in vitro*, aiming for potential application in future *in vivo* experiments. To conduct the experiment, a total of 240 previously disinfected spinach seeds (var. Crosstrek F1) were immersed in aqueous EO solutions for 10 minutes, then air-dried on sterile filter paper (Whatman no. 1) under controlled, sterile conditions at room temperature. For each treatment, sets of 15 seeds were placed on top of two layers of filter paper in 140 mm petri plate, ensuring uniform spacing. Petri plates were pre-moistened with 2 ml sterile distilled water (SDW), and covered with lids. Petri plates containing only distilled water served as the control group. The set up was kept in the growth chamber under suitable consistent conditions (85–90% RH, 20°C, and 12 h photoperiod). The experiment followed a completely randomized design (CRD) with four replicates. Seeds were considered germinated once 1 mm of the radicle had protruded through the seed coat. The seed germination percentage was evaluated seven days post-treatment according to the equation stated below.


$$Germination\;percentage\;\left(\%\right)\;=\frac{Total\;number\;of\;germinated\;seeds}{Total\;number\;of\;seeds}\;X\;100$$

### *Fusarium* wilt control

Two types of pot experiments were conducted to assess the effectiveness of promising EOs in mitigating *Fusarium* wilt. These experiments involved treatments applied to both seeds and soil, enabling a comprehensive evaluation of their impact on disease development. As mentioned earlier, fungal inoculum was incorporated into the soil at a rate of 1%, thoroughly mixed, and filled into sterile plastic pots two weeks prior to sowing. For the seed treatment, EOs were prepared in conical flasks at concentrations of 3.33 and 6.66 µL/mL. Spinach seeds were immersed in the prepared oils and shaken for 10 minutes using a shaker to ensure thorough saturation. Following this, the seeds were air-dried and planted with four seeds per pot (0.5 L). In the control treatment, seeds were soaked in SDW. Moreover, the same concentrations of these EOs, as used for seed treatment, were applied for soil treatment. During the planting process, the oils (15 ml) were initially added to each planting hole until saturation. As a control treatment, SDW was employed prior to planting. Pots were subjected to a temperature of 21°C and a photoperiod of 12 h in the growth chamber for a period of four weeks to monitor the development of *Fusarium* wilt. Disease incidence was evaluated using the aforementioned 0-3 scale for foliar symptoms, and subsequently the disease incidence reduction (DIR) was determined by using the following formula:


$$DIR\;\left(\%\right)\;=\;\frac{\left[DSI\;\left(control\right)\;-\;DSI\;\left(treatment\right)\right]}{DSI\;\left(control\right)}X\;100\;$$

The values calculated for DSI on the assessment day were used to compute the DIR in the respective experiment. Additionally, daily assessments were conducted over a period of 14 days post-sowing to determine the seedling emergence rate index (ERI), using the formula outlined by the Association of Official Seed Analysts (AOSA) (2002):


Emergence index=∑(Gt/Dt)

where *Gt* is the number of the germinated seed on day *t* and *Dt* is time corresponding to *Gt* in days. The irrigation process was performed as necessary, and all experiments were repeated twice.

### Statistical analyses

All experiments followed a randomized complete block design (RCBD), and the data are presented as means ± standard deviations (SD) and visually represented using box plots. Due to the lack of normality (Shapiro-Wilk test) and homogeneity of variances (Levene’s test), non-parametric hypothesis testing, specifically the Kruskal-Wallis test, was employed to assess the statistical significance of differences between treatments. In cases where significant differences were observed (p< 0.05), pairwise comparisons of the groups were conducted using Dunn’s test in the “R” software package (version 2.15.3). Probit analysis was utilized to calculate the ED50 values based on the OD values.

## Results

### Agar well diffusion

**Figure 1:**
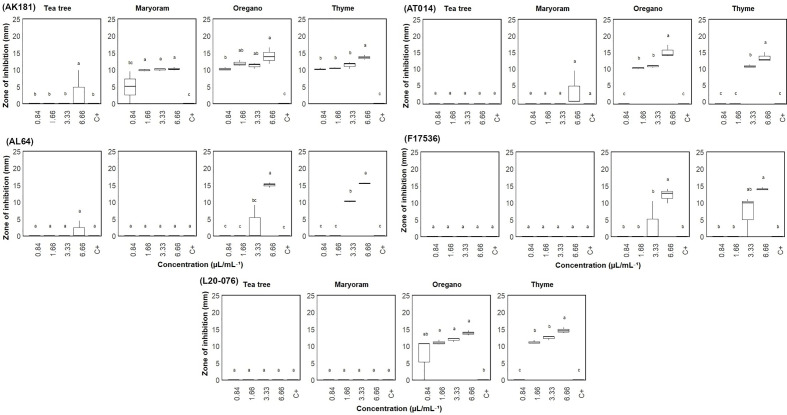
Zone of inhibition (mm) of *Fusarium* sp. measured via the agar well diffusion test on plates treated with varied concentrations (0.84-6.66 µL/mL) of essential oils. C+: Sterile distilled water served as positive control. Vertical bars represent the standard error (SE) of mean values of six replicates each including 2 wells. Boxes sharing the identical letters indicate values that do not differ significantly based on Dunn’s test (P > 0.05).

A sensitivity assessment using a well diffusion assay evaluated the response of five *Fusarium* strains (AL64, AK181, F17536, AT014, and L20-076) to four different EOs at concentrations ranging from 0.83 to 6.66 μL/mL, with clearance zones measured to determine their effects, as shown in **Figure 1**. The susceptibility of the selected fungal strains varied (P< 0.05) across different concentrations of EOs. Thyme and oregano EOs exhibited the highest level of antifungal activity among the tested oils. Marjoram also exhibited effectiveness, albeit to a lesser extent, by inhibiting only one strain (AK181). For strains AL64, L20-076, and F17536, thyme EO displayed the broadest inhibition zones, with average diameters of 15.46, 14.54, and 14.01 mm, respectively, followed by oregano at the highest concentrations (6.66 µL/mL). Similarly, for strains AT014 and AK181, oregano EO produced the widest inhibition zones of 15.33 and 14.01 mm (at 6.66 µL/mL), respectively, followed by thyme. Overall, among *Fusarium* strains, thyme and oregano EOs exhibited heightened antifungal activity in most cases, with AK181 emerging as particularly susceptible to marjoram. Conversely, marjoram and tea tree EOs demonstrated minimal variations in sensitivity across the tested strains, with some strains showing little to no response.

### Microtiter plate

**Figure 2:**
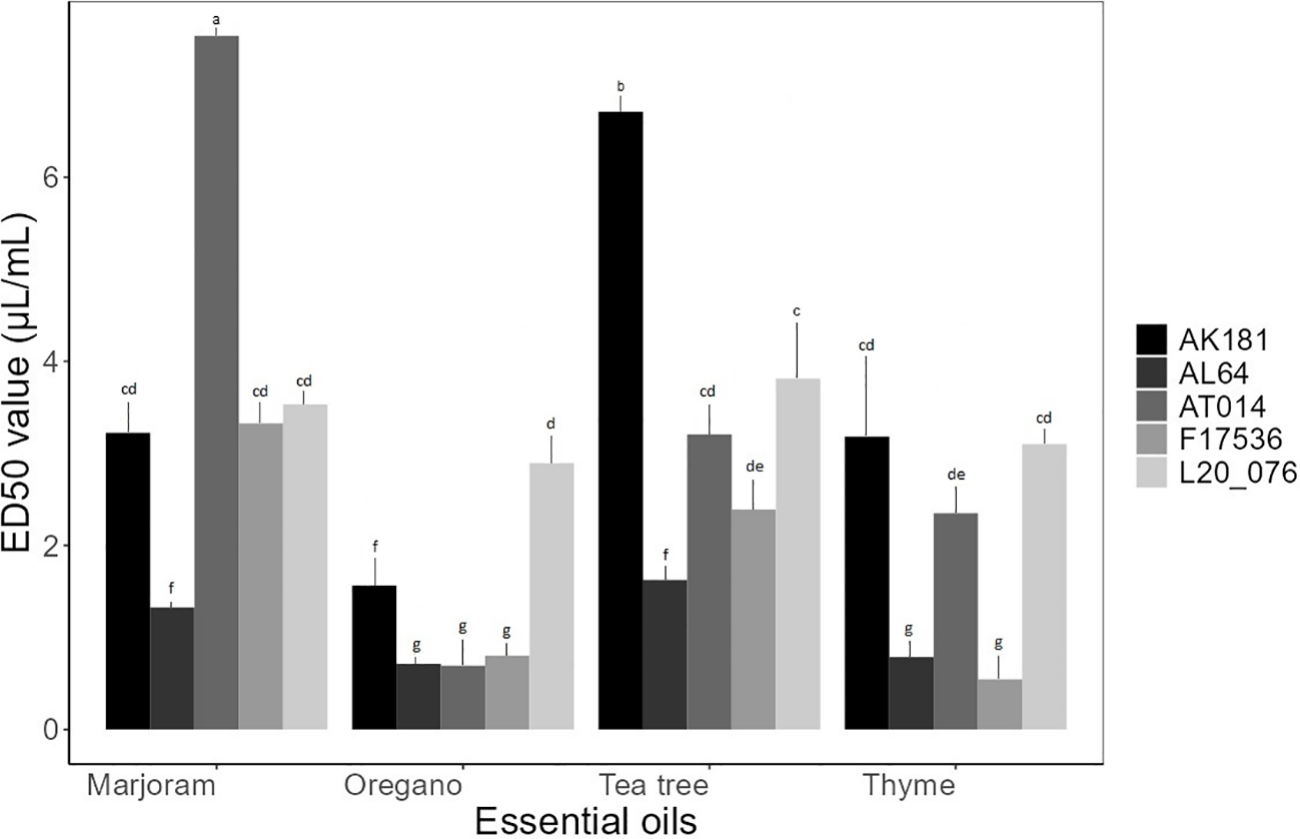
Mean effective dose (μL/mL) of essential oils for spore germination of *Fusarium* strains. Bars sharing the identical letters indicate values that do not differ significantly based on Dunn’s test (P > 0.05).

The baseline assay investigating the impact of four EOs on spore germination of *Fusarium* strains unveiled a spectrum of ED50 values ranging from 0.69 to 7.53 µL/mL. The statistical analysis revealed significant variations (p< 0.05) in the ED50 values among the tested compounds, indicating highly significant effects of different EOs on the response of various strains (**Figure 2**). Thyme and oregano EOs consistently exhibited lower ED50 values, indicating robust antifungal properties, while other EOs demonstrated varying levels of efficacy. Tea tree displayed moderate activity, with ED50 values ranging from 1.62 to 6.71 µL/mL across different *Fusarium* strains. In contrast, marjoram EO generally yielded higher ED50 values, averaging 3.78 µL/mL, suggesting comparatively lower effectiveness against spore germination. Notably, it exhibited an ED50 value of 7.53 µL/mL against AT014, indicating its weaker inhibitory effect in this particular instance.

### Agar dilution

**Figure 3:**
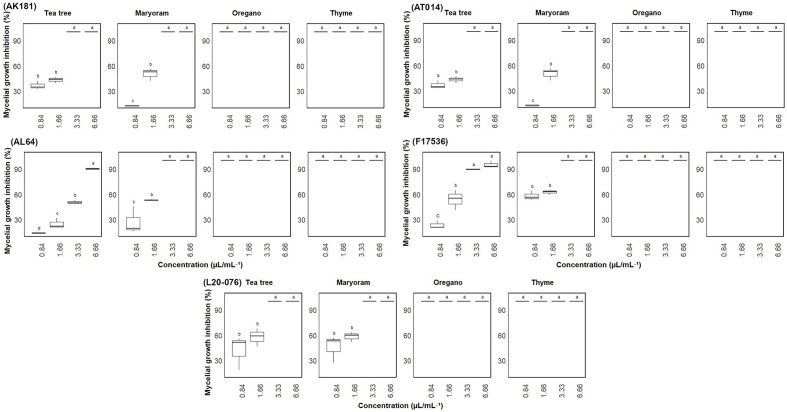
Mycelial growth inhibition (%) of *Fusarium* sp. on media amended with varied concentrations (0.84-6.66 µL/mL) of essential oils. Vertical bars represent the standard error (SE) of mean values of six replicates for each compound concentration. Boxes sharing the identical letters indicate values that do not differ significantly based on Dunn’s test (P > 0.05).

The inhibitory effect of EOs on the mycelial growth of various *Fusarium* strains was also investigated, revealing a dose-dependent response. As the concentration of EOs increased, the antifungal activity notably improved, resulting in inhibition of mycelial growth ranging from approximately 14% to 100% after seven days of incubation (**Figure 3**). All tested *Fusarium* strains exhibited similar sensitivity to the EOs. Notably, thyme and oregano EOs demonstrated complete inhibition (100% mycelial inhibition) against all *Fusarium* strains, even at the lowest concentration of 0.84 µL/mL. Marjoram EO also exhibited significant (p< 0.05) antifungal activity, with complete growth inhibition observed at concentrations of 3.33 and 6.66 µL/mL, while even at a lower concentration of 1.66 µL/mL, it considerably reduced mycelial growth of most tested strains. In contrast, tea tree EO displayed the weakest inhibitory effect, failing to entirely suppress mycelial growth in some strains, particularly AL64 and F17536, even at its maximum concentration of 6.66 µL/mL.

Furthermore, the assessment of fungistatic activity revealed that tee tree and marjoram EOs were effective at inhibiting the fungal growth at a concentration of 3.33 µL/mL. Thyme and oregano oils, however, displayed this activity even at 0.84 µL/mL, unlike the other EOs (**Table 1**). Indeed, tee tree and marjoram exhibited the highest MICs, indicating a lack of fungicidal impact on the fungal strains. Thyme EO displayed fungicidal activity ranging between 1.66 and 6.66 μL/mL, while Oregano ranged from 0.84 to 6.66 μL/mL across all strains tested. However, the importance of the surfactant Tween 80 added to the growth medium should not be overlooked, as it facilitated the even dispersion of the EOs, resulting in reducing the risk of inaccurately elevated MICs and MFCs. Overall, thyme and oregano consistently demonstrated the highest effectiveness among the four EOs, with a MIC of 0.84 µL/mL against nearly all tested strains in this study.

**Table 1:** Minimum inhibitory concentrations (MIC) and minimum fungicidal concentrations (MFC) of tee tree, thyme, oregano, and marjoram EOs (µL/mL) against the tested *Fusarium* strains.

Fusarium sp.	Tee tree		Thyme		Oregano		Marjoram	
	MIC	MFC	MIC	MFC	MIC	MFC	MIC	MFC
AL64	ND	ND	0.84	1.66	0.84	1.66	3.33	ND
AK181	3.33	ND	0.84	1.66	0.84	1.66	3.33	ND
F17536	ND	ND	0.84	1.66	0.84	1.66	3.33	ND
AT014	3.33	ND	0.84	1.66	0.84	0.84	3.33	ND
L20-076	3.33	ND	0.84	1.66	0.84	0.84	3.33	ND

ND, not detected.

### Volatile

**Figure 4:**
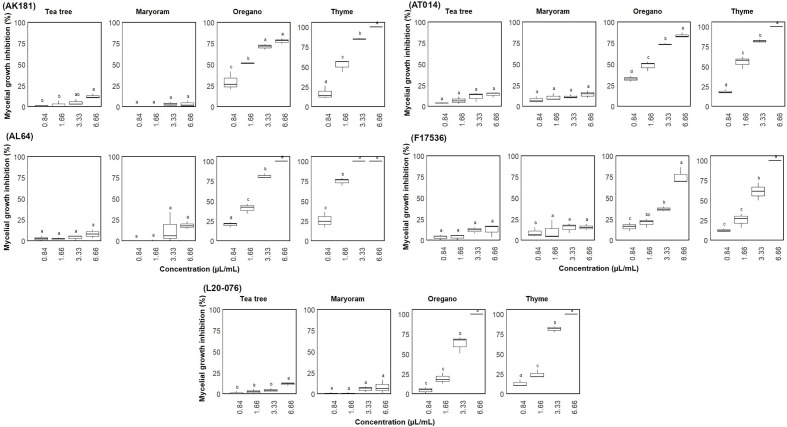
Mycelial growth inhibition (%) of *Fusarium* sp. on media through exposure to different concentrations (0.84-6.66 µL/mL) of essential oils. Vertical bars represent the standard error (SE) of mean values of six replicates for each compound concentration. Boxes sharing the identical letters indicate values that do not differ significantly based on Dunn’s test (P > 0.05).

The antifungal activity of tea tree, thyme, oregano, and marjoram EOs was investigated to detect their indirect effects in the vapor phase against *Fusarium* strains (**Figure 4**), contrasting with direct contact in the agar dilution method. Our results indicate that tea tree and marjoram oils had minimal to negligible effects on all strains, with mycelial growth inhibition consistently below 20% across all concentrations. In contrast, thyme and oregano notably EOs triggered complete growth inhibition in AL64 and L20-076 when applied at the highest concentration tested (6.66 µL/mL). Furthermore, volatiles from these oils consistently (P< 0.05) inhibited growth by over 80% across all strains at this concentration. Notably, the volatile compound derived from thyme exhibited the highest inhibitory potency, effectively halting the mycelial growth of at least one strain (AL64) at a concentration of 3.33 µL/mL.

Determining the lowest EO concentration resulting in 100% mycelial growth inhibition classified whether the effect was fungicidal or fungistatic. Fungistatic characterization involved observing growth upon transfer from the amended PDA plates to fresh PDA, while the absence of regrowth on fresh PDA indicated fungicidal activity. In this assay, thyme and oregano EOs, particularly at their highest concentration (6.66 µL/mL), exhibited varying degrees of antifungal activity. They demonstrated fungistatic effects against the tested *Fusarium* strains, except for AL64 and L20-076, where they showed a fungicidal effect (**Table 2**). Notably, thyme at a concentration of 3.33 µL/mL also displayed fungicidal activity against AL64. Additionally, while these oils effectively inhibited mycelial growth by 100% at the higher concentration, they did not demonstrate fungicidal or fungistatic effects on the *Fusarium* strains at lower concentrations. After comprehensive assessment of the outcomes derived from the *in vitro* bioassays, it was concluded that thyme and oregano EOs exhibited superior antifungal efficacy against *Fusarium* strains, particularly at concentrations of 3.33 and 6.66 µL/mL, prompting their selection for subsequent investigation in *in vivo* bioassays.

**Table 2:** Fungicidal or fungistatic effects of volatiles from thyme and oregano essential oils against the tested *Fusarium* strains.

Fusarium sp.	Thyme (µL/mL)		Oregano (µL/mL)	
	3.33 *	6.66	3.33	6.66
AL64	–	–	ND	–
AK181	ND	+	ND	+
F17536	ND	+	ND	+
AT014	ND	+	ND	+
L20-076	ND	–	ND	–

+, Fungistatic effect as mycelial regrowth observed on fresh PDA; -, fungicidal effect; ND, not detected.

*: Concentrations of thyme and oregano essential oils as µL/mL.

### Molecular identification of *Fusarium* strains

To strengthen confidence in strains identification and minimize the risk of result misinterpretation, we amplified the ITS rDNA and TEF-1α regions of all isolates. The BLAST search showed the PCR products from three major *Fusarium* species including *F. equiseti, F. oxysporum*, and *F. incarnatum-equiseti* exhibited a 99-100% homology with the associated sequences in the GenBank database (**Table 3**). Our analysis identified three strains belonging to the *F. oxysporum* species complex (L20-076, AS-109, and L20-080), three strains belonging to *F. equiseti* (AT-014, L20-92 and L21-014), one strain belonging to *F. oxysporum* f.sp. *cepae* (AK-181), one strain belonging to the *F. incarnatum-equiseti* species complex (AL-64), and three strains belonging to *F. oxysporum* f.sp. *spinaciae* (F-100.2, F14157 and F-17536). Consequently, we selected a single strain of *F. oxysporum* f.sp. *spinaciae* (F-17536) for *in planta* experiments involving spinach, considering its relevance to the target plant and its prior assessment in *in vitro* experiments.

**Table 3:** Molecular identification of 11 *Fusarium* strains obtained from different sources.

Strain	Fungal taxa	Host	Provider^*^
AT-014	*F. equiseti*	Spinach	Enza Zaden
L20-076	*F. oxysporum*	Leafy green	Enza Zaden
AS-109	*F. oxysporum*	Spinach	Enza Zaden
L20-080	*F. oxysporum*	Leafy green	Enza Zaden
L20-192	*F. equiseti*	Spinach	Enza Zaden
L21-014	*F. equiseti*	Lamb lettuce	Enza Zaden
AK-181	*F. oxysporum* f.sp. *cepae*	Leafy green	Enza Zaden
AL-64	*F. incarnatum-equiseti*	Lamb lettuce	Enza Zaden
F-100.2	*F. oxysporum* f.sp. *spinaciae*	Spinach	CREA
F-14157	*F. oxysporum* f.sp. *spinaciae*	Spinach	CREA
F-17536	*F. oxysporum* f.sp. *spinaciae*	Spinach	CREA

^*^ Enza Zaden: Vegetable Breeding Company, Netherlands; CREA; Research Centre for Vegetable and Ornamental Crops, Italy.

### Assessments of the antifungal activity of EOs - in planta

#### Inoculation method

**Figure 5:**
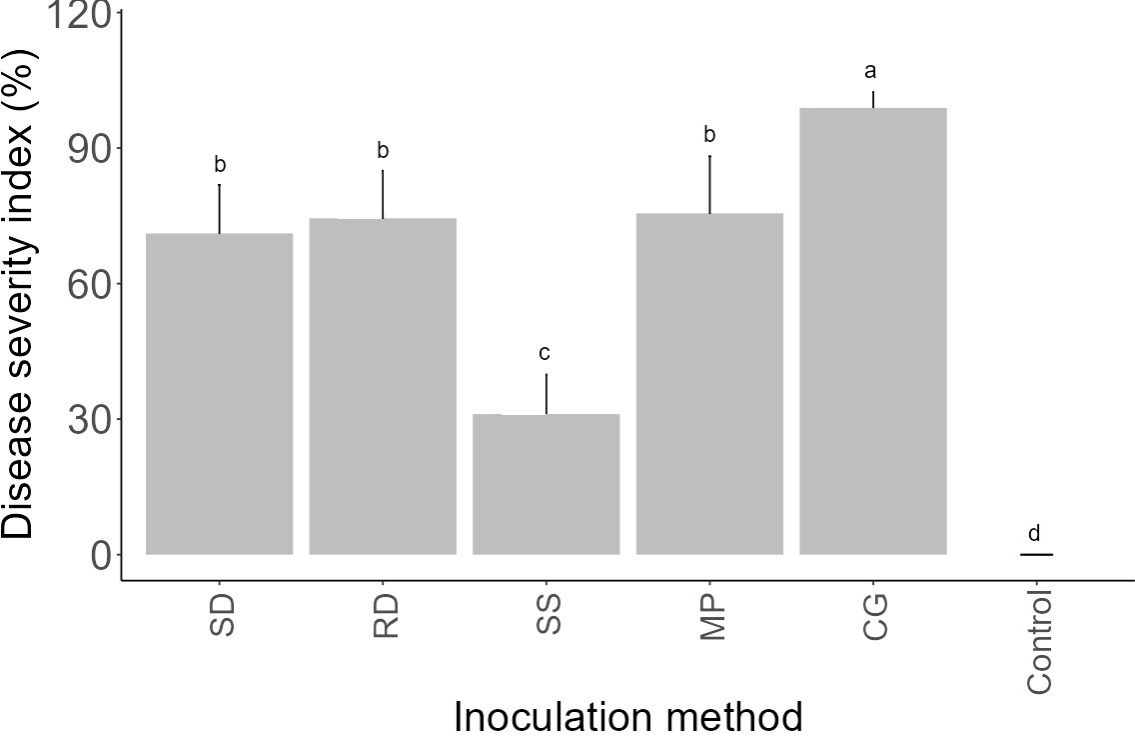
Mean *Fusarium* wilt severity index (%) on spinach (var. Crosstrek F1) induced by *F. oxysporum* f.sp. *spinaciae* 22 days post-inoculation using five distinct inoculation methods. SD: soil drenching; RD: root dipping; SS: seed soaking; MP: mycelial plug; CG: colonized grain. Vertical bars represent the standard error (SE) of mean values of 10 pots each including 3 plants. Barplots sharing the identical letters indicate values that do not differ significantly based on Dunn’s test (P > 0.05).

To ascertain the most efficient approach for greenhouse-based inoculation, an assessment was conducted on five distinct inoculation methods, namely SD, RD, SS, MP, and CG. This evaluation was performed on a specific strain of *F. oxysporum* f.sp. *spinaciae* (F-17536) following preliminary *in vitro* bioassays and considering its original host plant. All five inoculation techniques induced symptoms of *Fusarium* wilt in the spinach plants, resulting in significantly varied levels of disease severity (P< 0.05) (**Figure 5**). Reddening attributed to the presence of fungal mycelium was observed alongside the root vascular tissue in infected seedlings, which exhibited a water-soaked appearance or brown to black discoloration. All inoculation methods displayed initial disease symptoms approximately 14 days after inoculation (DAI), except for the CG method, which manifested symptoms at 9 DAI, characterized by poor germination, stunted growth, and post-emergence damping off. However, the RD, SD, and MP methods displayed comparable levels of disease severity which were significantly (P< 0.05) different from the control. Though the SS method did induce mild foliar symptoms, it was inconsistent throughout the experiments. All of the non-inoculated control plants remained asymptomatic over the duration of each pathogenicity test. As a general observation, the effectiveness of inoculating methods could be arranged in the following order: CG method exhibited the highest efficacy, succeeded by methods MP, RD, SD, and SS, respectively. Consequently, the GC method was selected for conducting further *in vivo* experiments.

### Phytotoxic effect

The impact of thyme and oregano EOs on the germination rate of spinach seeds is presented in **Table 4**. By comparing the average germination rates of different treatments, including various concentrations of EOs, with those of the control group, we could determine if there were any harmful effects on seed germination. At 6.66 µL/mL, oregano oil showed a marginal 2% decrease in germination percentage compared to the control, which is practically negligible and might be compensated for by increasing the seed amount. Thyme oil at 3.33 and 6.66 µL/mL and oregano oil at 3.33 µL/mL did not affect germination rates, indicating no phytotoxic impact of treatments on seed germination across all concentrations used when compared to the untreated control.

**Table 4:** Percentage of germination rate on day seven after treatment of spinach seeds with thyme and oregano essential oils.

Thyme (µL/mL)	Germination rate(%)		Oregano (µL/mL)	Germination rate (%)	
0	97 ± 2.0	a	0	97 ± 2.0	a
3.33	97 ± 2.0	a	3.33	97 ± 3.82	a
6.66	97 ± 3.82	a	6.66	95 ± 2.0	a

Means (± SD) sharing the identical letter in a column denote no significant differences compared to the untreated control (0 µL/mL), according to Dunn’s test (P > 0.05).

### *Fusarium* wilt control

In this phase of our study, the primary aim was to mitigate *Fusarium* wilt in spinach seedlings through the application of two efficient EOs candidates in both seed and soil treatments. Focusing on oregano and thyme at concentrations of 3.33 and 6.66 µL/mL, we sought to evaluate the impact of these treatments on disease incidence and the emergence rate of seedlings. There was no significant difference (p > 0.05). in the mean ERI values 14 days after sowing spinach seeds treated with EOs compared to the untreated control (**Table 5**). Soil treatment with varying concentrations of EOs also resulted in no significant difference in the ERI values, indicating that the pathogen was not able to interfere the development of spinach seeds. Additionally, a clear relationship found between the application of these EOs and their concentrations in terms of DIR. Indeed, thyme and oregano EOs consistently reduced disease occurrence across various concentration levels (P< 0.05), with no discernible impact on seeds emergence. For instance, thyme, in seed treatment at 6.66 µL/mL, exhibited an ERI of ≈ 7 and the highest DIR of ≈ 54%. The remarkable performance of thyme emphasizes its potential for effective disease control in spinach seedlings, making it a promising candidate for further exploration in *Fusarium* wilt management strategies. The data from the experiment also revealed that seed treatments with thyme and oregano EOs at various concentrations consistently led to considerable DIR compared to soil treatments, highlighting the superior efficacy of seed-based interventions in controlling disease occurrence. In essence, using EOs as seed dressing proved more effective than soil treatment, with thyme oil showing the most promising outcomes among the oils tested.

**Table 5:** Emergence rate index and reduction of *Fusarium* wilt incidence in spinach seedlings through the application of oregano and thyme EOs in seed and soil treatments.

Essential oil	Concentration (µL/mL)	Seed treatment		Soil treatment	
		ERI	DIR (%)	ERI	DIR (%)
Oregano	3.33	$7.7\pm 0.79{\;}_{\text{a}}^{\text{a}}$	$16.66{\;}_{\text{c}}^{\text{a}}$	$6.34\pm 1.69{\;}_{\text{a}}^{\text{a}}$	$8.33\pm 9.62{\;}_{\text{c}}^{\text{b}}$
	6.66	$5.77\pm 3.56{\;}_{\text{a}}^{\text{a}}$	$36.11\pm 5.55{\;}_{\text{b}}^{\text{a}}$	$5.24\pm 2.68{\;}_{\text{a}}^{\text{a}}$	$16.66\pm 13.6{\;}_{\text{a}}^{\text{b}}$
Thyme	3.33	$6.34\pm 0.96{\;}_{\text{a}}^{\text{a}}$	$30.55\pm 5.55{\;}_{\text{b}}^{\text{a}}$	$6.71\pm 2.25{\;}_{\text{a}}^{\text{a}}$	$12.5\pm 15.95{\;}_{\text{b}}^{\text{b}}$
	6.66	$7.07\pm 2.02{\;}_{\text{a}}^{\text{a}}$	$54.16\pm 9.48{\;}_{\text{a}}^{\text{a}}$	$7.8\pm 1.38{\;}_{\text{a}}^{\text{a}}$	$18.05\pm 2.77{\;}_{\text{a}}^{\text{b}}$
	0	$6.69\pm 0.71{\;}_{\text{a}}^{\text{a}}$	$0{\;}_{\text{d}}^{\text{a}}$	$7.06\pm 1.29{\;}_{\text{a}}^{\text{a}}$	$0{\;}_{\text{d}}^{\text{a}}$
CV (%)		21.48	12.77	29.52	85.04

DIR, Disease incidence reduction in percentage; ERI, Emergence rate index; CV, Coefficient of variation in percentage. Values represent mean ± SD. Identical superscript letters within a row denote no significant differences (P > 0.05), Duncan’s multiple-range test) in each concentration for the same treatment. Different subscript letters within a column denote significant differences (p< 0.05), Duncan’s multiple-range test) between concentrations for the same treatment.

## Discussion

In response to the growing need to reduce reliance on chemical substances for plant protection and combat resistance to existing chemicals, there has been a notable increase in the investigation of natural-based compounds. This aims to alleviate the adverse impacts of synthetic pesticides on human health and the environment. Additionally, spinach, an important leafy green crop, is predominantly threatened by soilborne diseases that target its vascular tissue, posing a considerable challenge to its cultivation worldwide. Therefore, the current study aimed to assess antifungal activity of certain plant-derived EOs through both *in vitro* and *in planta* experiments, with the overarching objective of managing *Fusarium* wilt disease in spinach. Fungal growth was influenced by all the tested EOs, although the degree of inhibition differed depending on the specific oil and the strains investigated. The EOs utilized in our study, commercially produced, are known for their effectiveness as biocontrol agents against various phytopathogens. Also, their extraction through standardized procedures enhances the reliability and repeatability of outcomes. Nevertheless, aside from environmental factors, inconsistencies in the efficiency of EOs may occur as a result of their extraction from different plant parts and under varying laboratory conditions, as indicated in previous studies (Dan et al., 2010; Amini et al., 2016; Sarkhosh et al., 2018; Najdabbasi et al., 2020).

We found notable efficacy in inhibiting spore germination and mycelial growth across strains for both thyme and oregano EOs. Oregano EO revealed the most extensive inhibition zone and consistently lower ED50 values, highlighting its potent antifungal attribute. Following oregano, thyme EO also showed considerable effectiveness, while other EOs demonstrated varying levels of efficacy against the tested strains. Such inhibitory effects could be linked to the enzymatic activity of the EOs by disrupting cell wall structure and impeding membrane synthesis (Rasooli et al., 2006; Li et al., 2014). In a study by Parikh et al. (2021), the inhibitory effects of 38 EOs on 10 important pathogenic fungi and oomycetes were examined, revealing that thyme, clove, lemongrass, cinnamon, and oregano EOs exhibited the most potent inhibitory effects on spore germination, sporangia production, and mycelial growth of the pathogens. However, the levels of inhibitory effect varied among the different plant pathogens. In our investigation, thyme and oregano EOs could also effectively suppress *Fusarium* growth through fungicidal activity, at concentrations exceeding 0.84 μL/mL, as opposed to the fungistatic effects observed with tea tree and marjoram at 3.33 µL/mL. Diverse volatile bioactive compounds such as tannins, carvacrol, flavonoids, borneol, sterols, quinones, saponins, alkaloids, and phenols present in EOs cause a range of biological activities, including antibacterial, insecticidal, antiviral, and antifungal properties (Daferera et al., 2003; Kim et al., 2003; Burt, 2004; Schnitzler et al., 2007; Halmer, 2008; Kerekes et al., 2013; Nazzaro et al., 2017). For instance, the antifungal capability of oregano oil may be linked to the presence of its primary components, carvacrol and thymol, which are phenolic compounds known for their antioxidant effects (Mechergui et al., 2010). Utilizing GC-MS analysis, Soylu et al. (2006) also identified 16 bioactive compounds in thyme oil, with carvacrol constituting the major component at 37.9%. Considering the significant presence and activity of phenols and monoterpenic alcohols, it was not surprising that oregano and thyme oils were among the most effective in inhibiting all tested *Fusarium* strains in our study, consistent with previous research (Kishore et al., 2007; Regnier et al., 2014; Dewitte et al., 2018; Najdabbasi et al., 2020). In our study, a uniform level of MFC values exhibiting fungicidal activity was observed for most strains, with the exception of oregano, which showed reduced activity for two *Fusarium* strains (AT014 and L20-076). EOs with the lower MFCs are not only more effective and preferable for disease management but are also desirable for reducing both the volume and cost of applications. On the contrary, their efficacy without fungicidal activity might be weakened upon initial contact with the pathogen, especially through evaporation and washing away (Parikh et al., 2021).

To assess the possible phytotoxic impact of the chosen EOs on germination, spinach seeds were subjected to seed treatments with the EOs. Indeed, treating seeds to prevent soil-borne pathogens when planting leafy green vegetables is a widespread disease management practice. In our study, a standard germination rate exceeding 90% was observed across all treated seeds, affirming that even at a concentration of 6.66 µL/mL, these oils do not exhibit phytotoxic effects on spinach seeds germination. Similar findings were observed by Parikh et al. (2021), where EOs derived from oregano, clove, cinnamon, palmarosa, and thyme did not affect the germination of chickpea seeds, with a germination rate of 90 to 95%, comparable to that of untreated control seeds. Orzali et al. (2020) also reported no influence of *Origanum vulgare* EO on the germination rate of tomato seeds, consistent with the findings of Flores et al. (2018), who investigated the antibacterial efficacy of oregano, thyme, and cinnamon EOs without observing any phytotoxic effects on tomato seedlings. However, the phytotoxic impacts of EOs on various crop seeds have been previously documented (Mahdavikia and Saharkhiz, 2015; Boukaew et al., 2017; Ibáñez and Blázquez, 2018; Perczak et al., 2019; Jouini et al., 2020), which are mainly associated with allelopathy, a non-selective phenomenon that becomes serious only if seeds or plants possess enzymatic mechanisms of eliminating toxicogenic potential (Sumalan et al., 2019; Benarab et al., 2020; Bota et al., 2022). Overall, the type and concentration of the EO, along with the plant species exposed to it, determine the phytotoxic level (Somda et al., 2007; Orzali et al., 2014),. Hence, establishing the ideal concentration of EOs possessing potent antifungal properties yet are free of phytotoxicity is always crucial.

To delve deeper into controlling *Fusarium* wilt in greenhouse settings, we initially assessed different inoculation methods employing a specific strain of *F. oxysporum* f.sp. *spinaciae*, F-17536, to induce the infection in spinach, aiming to establish a reliable and reproducible methodology. An optimal inoculation technique guarantees effective colonization of the host plant, enabling a thorough disease assessment. In the comparison of the five inoculation methods, we suggest seeding spinach in a mixture of *Fusarium*-colonized ground wheat (GC) and substrate at a ratio of 1%. As the preferred protocol for *Fusarium* wilt screening, we effectively exposed spinach to a concentrated pathogen load, thereby intensifying disease pressure from the soil. This resulted in symptoms appearing as early as 9 DAI, in contrast to the 14 DAI observed with other inoculation methods. However, the presence of conidia in the mycelia might have interfered with this outcome. This inoculation method also simulates the presence of soil-borne fungal inoculum in natural field conditions, representing a primary source of infections (Hörberg, 2002; Hoheneder et al., 2022). Lai et al. (2020) showed that introducing *Fusarium*-colonized barley inoculum during sugar beet seed planting led to the development of more severe disease symptoms in a shorter timeframe compared to the root-dipping technique. Although the colonized grain-based inoculum technique has been demonstrated as suitable for studying various soil-borne pathogens and host plants (Holmes and Benson, 1994; Bai and Shaner, 2004; Kirk et al., 2008; Mirmajlessi et al., 2012; Zhang et al., 2014; Noor and Khan, 2015; Koch et al., 2020; Lai et al., 2020; Hoheneder et al., 2022), it was applied for the first time to test the pathogenicity of *F. oxysporum* f.sp. *spinaciae* on spinach, leveraging strengths from previous studies. The disease symptom induced by MP, RD, and SD inoculation methods also led to comparable severe *Fusarium* infections. The observed uniformity in DSI among these three methods is likely because of their shared capacity to infiltrate the root epidermis and colonize tissues intracellularly and intercellularly, as reported by previous studies (Van Peer and Schippers, 1992; Mendgen et al., 1996; Czymmek et al., 2007; Lai et al., 2020). However, despite their effectiveness, these methods are time consuming and resource-intensive, requiring large quantities of inoculant and nursery preparation. In contrast, the SS method was found to be less effective, likely due to factors such as limited survival of the inoculant, inadequate spore quantities on seeds, or the removal of attached spores during watering. Nonetheless, it offers cost savings for large-scale experiments as it needs low amount of inoculant compared to other methods (Rocha et al., 2019).

Continuing with greenhouse experiments, we evaluated the effects of the most potent EOs (thyme and oregano), determined *in vitro*, on the *F. oxysporum* f.sp. *spinaciae*-spinach pathosystem *in vivo*, laying the groundwork for future field experiments. Since greenhouse experiments can be adjusted to mitigate environmental variables, creating optimal growth conditions and gaining fresh insights into disease management within a shorter time frame, they can yield comparable assessments to field evaluations, as reported by previous studies (La Torre et al., 2016; Borrero et al., 2017; Gonçalves et al., 2021). For instance, Ribeiro et al. (2015) found that while field evaluations for testing genotype resistance to *Fusarium oxysporum* f. sp. *cubense* isolates typically span two years, greenhouse experiments could achieve similar assessments in approximately three months. We observed that at the highest concentration, thyme oil exhibited greater efficacy against *F. oxysporum* f.sp. *spinaciae* compared to oregano oil. This resulted in approximately 54% and 17% reductions in disease incidence on spinach in both seed and soil treatments, respectively, without causing any phytotoxic effects. Indeed, the antifungal activities of these EOs are primarily due to high levels of carvacrol and thymol, as reported in several studies (Bouchra et al., 2003; Neri et al., 2006; Combrinck et al., 2011; Pérez-Alfonso et al., 2012; Zhang et al., 2019). Carvacrol suppresses Cyclooxygenase (COX-2) expression and activates peroxisome proliferator-activated receptors (PPAR) α and γ (Hotta et al., 2010; Mączka et al., 2023), while thymol induces the production of reactive oxygen species (ROS) and lipid peroxidation (LPO) in fungal cells (Ma et al., 2019; Shcherbakova et al., 2021). The reduced efficacy of soil treatment may result from the uneven distribution of EOs in the soil matrix, leading to varying concentrations around treated seeds and limiting their effectiveness in inhibiting fungal growth. Besides, the interaction between EO and soil components can also affect the bioavailability and stability of active compounds, thereby reducing its antifungal activity. In contrast, immersing seeds in the EO could potentially create a protective obstacle against contaminated soil during the germination process. These could explain why *Fusarium* wilt was satisfactorily inhibited in seeds treated with relatively high concentrations of these oils, which is consistent with findings from other studies (Mbega et al., 2012; Karaca et al., 2017; Cadena et al., 2018; Terzić et al., 2023). Van Der Wolf et al. (2008) demonstrated the antifungal properties of thyme, oregano, cinnamon, and Clove EOs against various seed-borne pathogens, highlighting thyme as the most effective natural compound for mitigating infections in cabbage seeds. In a study conducted by Ben-Jabeur et al. (2015), thyme EO also revealed effective control of gray mold and *Fusarium* wilt in tomato seedlings. It resulted in a reduction of *Botrytis cinerea* colonization by 64% and significantly lowered *Fusarium* wilt severity by 30.76%, compared to the untreated control, especially seven days post-treatment. In practice, adjusting the treatment duration and dosage is crucial when applying oil to seeds before sowing to maximize antifungal action against soil-borne pathogens without causing phytotoxicity. Furthermore, the susceptibility of EOs to environmental factors like light, temperature, and humidity, along with their high volatility, can lead to instability, potentially affecting their effectiveness over time (Turek and Stintzing, 2013; Najdabbasi et al., 2020). To address these challenges, EOs can be integrated into novel formulations like microemulsions, nanoparticles, and encapsulation techniques, which enhance their physicochemical stability, reinforce biocidal effectiveness, and prolong their activity against plant pathogenic fungi and bacteria (Zhang et al., 2014; Pavoni et al., 2019; Weisany et al., 2019; Basavegowda et al., 2020; Tu et al., 2020; Weisany et al., 2022). For instance, Sivalingam et al. (2024) showed that essential oil-loaded nanostructured lipid carriers displayed antifungal activity against *Fusarium oxysporum* f.sp. *lycopersici*, comparable to the positive control carbendazim. Taken together, these aspects suggest that although seed treatment with EOs holds promise as a control strategy, improving application methods and formulations is essential to enhance their effectiveness in managing *Fusarium* wilt in spinach cultivation.

## Conclusion

This study collectively assessed various artificial inoculation methods to induce *Fusarium* wilt in spinach under greenhouse conditions. It found that the colonized grain-based inoculum method was the most effective approach in eliciting symptoms, which appeared at approximately 9 DAI, thus providing valuable insight for future *Fusarium* research on spinach. Furthermore, given that previous studies have primarily focused on other *Fusarium* species when assessing the fungitoxicity of EOs, our research provides additional evidence supporting the potential use of thyme and oregano oils for controlling *F. oxysporum* f.sp. *spinacia*. To our knowledge, this study is the first to investigate the application of these EOs for treating spinach seeds and soil in a greenhouse environment, resulting in a reduction in *Fusarium* wilt incidence without adverse effects on plant health. Supported by *in vivo* experiments, these findings highlight the potential of thyme EO as a green fungicide for integrated pest management in spinach cultivation. However, successful large-scale application of such compounds requires formulation optimization, suitable farm facilities, and consideration of various biological and environmental factors.

## Data availability statement

The raw data supporting the conclusions of this article will be made available by the authors, without undue reservation.

## Author contributions

MM: Conceptualization, Data curation, Investigation, Methodology, Supervision, Validation, Visualization, Writing – original draft, Writing – review & editing. NN: Data curation, Formal analysis, Investigation, Methodology, Software, Writing – original draft, Writing – review & editing. LS: Resources, Writing – review & editing. GH: Funding acquisition, Project administration, Resources, Writing – review & editing.
